# Insights into the structure of grey reef shark aggregation, *Carcharhinus amblyrhynchos* (Bleeker, 1856), in two distinct channels of the Maldivian archipelago, Indian Ocean

**DOI:** 10.1111/jfb.70337

**Published:** 2026-03-03

**Authors:** Andrea Parmegiani, Jacopo Gobbato, Rilwan Yoosuf, Davide Seveso, Paolo Galli, Andrew Darren Whitehead, Yameen Ismail, Simone Montano

**Affiliations:** ^1^ Department of Earth and Environmental Sciences (DISAT) University of Milano‐Bicocca, Piazza Della Scienza Milan Italy; ^2^ MaRHE Center (Marine Research and High Education Center) Magoodhoo Faafu Atoll Republic of Maldives; ^3^ White Wave Maldives PVT. LTD Malè City Republic of Maldives; ^4^ Environmental Regulatory Authority Dharubaaruge Malé Republic of Maldives; ^5^ NBFC (National Biodiversity Future Center) Palermo Italy; ^6^ University of Dubai Dubai United Arab Emirates; ^7^ Shark Research Mexico A.C La Paz Mexico

**Keywords:** biometric measurement, Carcharhiniformes, elasmobranch, key biodiversity area, photogrammetry, shark ecology

## Abstract

The grey reef shark *Carcharhinus amblyrhynchos* is one of the most frequently encountered reef‐associated shark species in the Maldives, yet very little is known about its local size structure or population dynamics. This study provides new insights into two aggregations of *C. amblyrhynchos* in the Maldives, based on biometric measurements obtained with laser photogrammetry, standardized visual surveys, opportunistic behavioural observations, and video analyses of citizen science data spanning 2013–2024. In particular, between 2022 and 2023, biometric data and visual observations from two distinct sites, Villingili Kandu in North Huvadhu Atoll and Miyaru Kandu in Vaavu Atoll, were collected to preliminarily characterize the composition of these aggregations and to provide information on the ecology of grey reef sharks. The results revealed a pronounced female‐biased sex composition and occurrences of mating in both channels, as well as a potential nursery area in Miyaru Kandu. Combining these data with predation and cleaning events recorded, we suggest that these channels might constitute important key habitats for grey reef shark aggregations. Laser photogrammetry data from 353 individuals were analysed, and a robust relationship between pre‐caudal length and total length (*R* ^2^ = 0.97) was established, enabling the derivation of a correction index through linear regression to predict and enhance the accuracy of size estimates. Finally, long‐term video records demonstrate that Villingili Kandu currently hosts the largest documented aggregation of *C. amblyrhynchos* in the Maldives. These initial findings provide a baseline for further studies on the drivers influencing the aggregations of this species and its life history within the archipelago.

## 1 INTRODUCTION

Shark‐related tourism has become a significant source of income for marine ecotourism in the Maldives, with revenues starting at approximately USD 2.3 million in 1991 (Anderson & Ahmed, 1993) and recently surpassing USD 51 million (Zimmerhackel et al., 2019). This economic growth demonstrates the significance of shark tourism compared to shark fishing activities, which, before the total shark ban of 2010, generated an estimated revenue of approximately USD 0.5 million (Anderson & Ahmed, 1993; Zimmerhackel et al., 2019). This comparison clearly demonstrates the shift in national priorities, from extractive exploitation to conservation‐driven economic strategies that benefit both biodiversity and local communities.

The rich biodiversity of elasmobranchs in the Maldives has played a pivotal role in the development of the diving sector, with reoccurring or resident aggregations of charismatic elasmobranch species driving these activities, such as reef manta rays (*Mobula alfredi*) (Armstrong et al., 2021; Harris et al., 2020; Harris & Stevens, 2021), whale sharks (*Rhincodon typus*) (Riley et al., 2010; Valsecchi et al., 2021) and other predatory species such as tiger sharks (*Galeocerdo cuvier*) (Vossgaetter et al., 2024) and bull sharks (*Carcharhinus leucas*) (Parmegiani et al. 2023). With the term aggregation, we refer to the co‐occurrence of two or more individuals in both space and time, generally facilitated by ecological or behavioural drivers (McInturf et al., 2023). The typical drivers that are fundamental for forming such aggregations may vary depending on the species and environmental context, with foraging being one of the most important (Ketchum et al., 2012; Sims & Victoria, 1998; Towner et al., 2016). In particular, sharks generally locate favourable conditions to forage or hunt together, as documented for *R. typus* in several places around the world (Andrzejaczek et al., 2016; Copping et al., 2018; La Parra Venegas et al., 2011; Norman et al., 2017; Perry et al., 2018; Robinson et al., 2013; Rohner et al., 2020; Whitehead et al., 2019). Other aggregation drivers include mating and reproductive behaviour, where females form sex‐segregated aggregations, such as *Carcharhinus falciformis* in Mexico (Whitehead et al., 2022), or aggregation defence mechanisms, usually found for *Carcharhinus limbatus* (Quoy and Gaimard, 1824), which effectively form groups for protection against predators (Doan & Kajiura, 2020). Furthermore, another driver for shark aggregations is the hydrodynamic efficiency, as sharks minimise energy expenditure through specific formations that reduce the energy outlay of both individual specimens and the group (Papastamatiou et al., 2021; Porter et al., 2020). More recently, *Carcharhinus amblyrhynchos* has also been observed forming aggregations at isolated reef ledges where individuals show resting behaviour, remaining largely unresponsive (Bullock et al., 2024).

One of the most frequently observed reef‐associated shark aggregations in the Maldives is composed of grey reef sharks, *C. amblyrhynchos*, along atoll channels or reefs (Clarke et al., 2012). However, at present, limited information is available regarding the local size structure or population dynamics of this species. The only published data consists of a report by Anderson et al. (1992). Despite the Maldives’ importance as a shark sanctuary in the Indian Ocean (Robinson et al., 2022), substantial gaps still remain in the scientific literature concerning shark behaviour, general ecology, and aggregation dynamics within different habitats. In particular, many known aggregation sites remain poorly studied or lack sufficient data to be considered for conservation initiatives from governmental entities. Recently, researchers from the IUCN Shark Specialist Group led the delineation of portions of habitats critical to several shark species as important shark and ray areas (ISRAs), of which 27 have been identified in the Maldives (Jabado et al., 2023); however, of the two areas surveyed in the present study, only one has been identified as an area of interest. Moreover, the increasing availability of underwater video footage and observations from recreational divers is enhancing possibilities of shark research, with citizen science providing broad spatiotemporal datasets that, once validated, represent a valuable tool for advancing ecological and behavioural studies (Araujo et al., 2020; Bargnesi et al., 2020; Gobbato et al., 2024).

In this context, our study aimed to evaluate and provide an overview of shark aggregations in these two key channel sites: Villingili Kandu, in Gaafu Alifu Atoll and Miyaru Kandu in Vaavu Atoll, the latter of which is a designated marine protected area (MPA) since 1995. Using in situ observational and photographic methods, the study focused on assessing the ecological importance of the two channels, obtaining biometric data and providing insights into the ecology of *C. amblyrhynchos*.

## 2 MATERIALS AND METHODS

### 2.1 Study site

**FIGURE 1:**
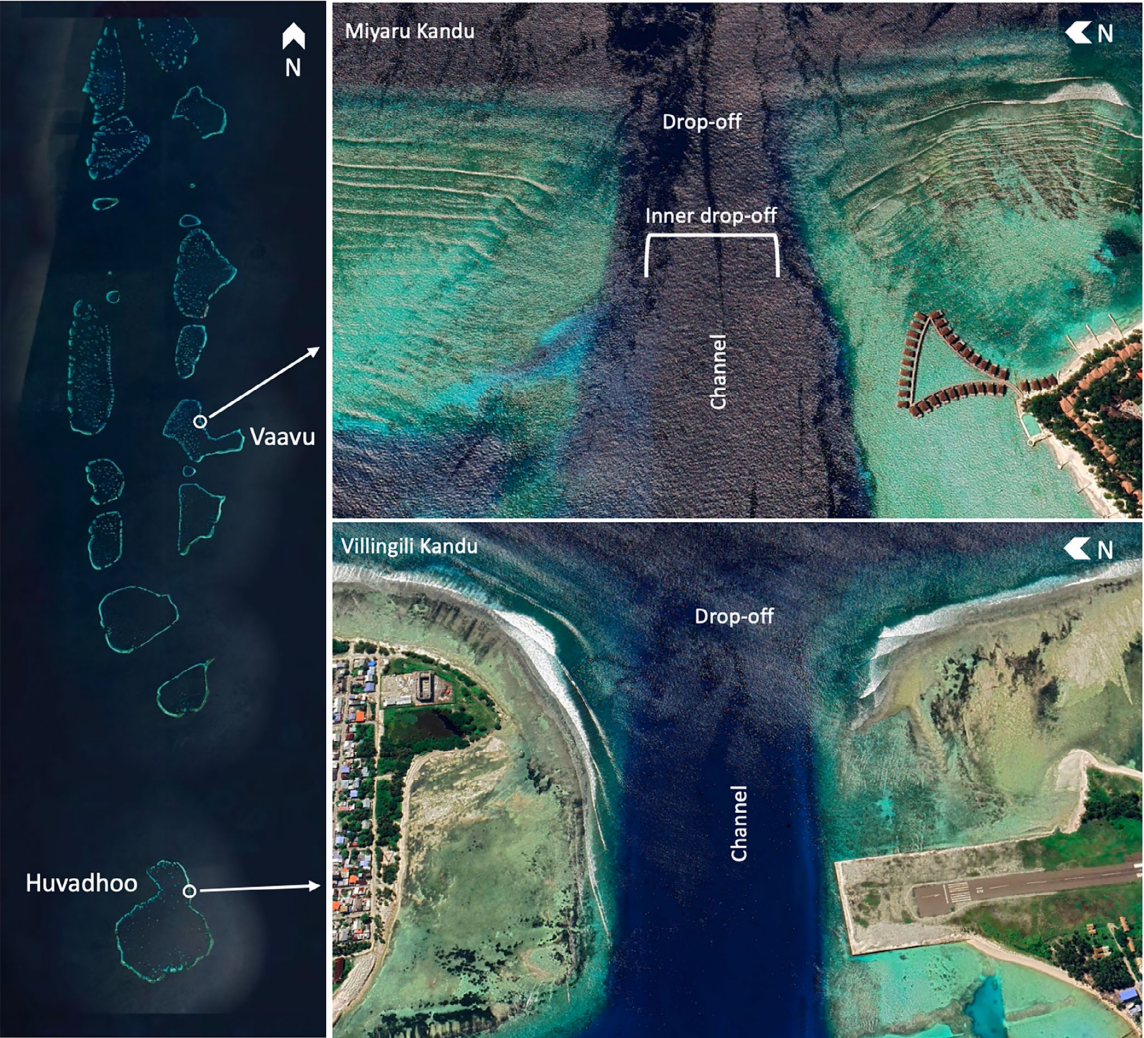
Overview of the study area of Miyaru Kandu and Villingili Kandu, the channels in Vaavu Atoll and Gaafu Alifu Atoll, Maldives. Map created by MapTiler and Google Earth Pro.

The study was conducted in two sites called Villingili Kandu and Miyaru Kandu. Kandu means ‘channel’ in the local Dhivehi language, indicating the strip of water between two islands at the edge of an atoll. In particular, Villingili Kandu is a channel situated in Huvadhu Atoll, in the southern region of the Maldives archipelago (Figure 1). The channel is flanked by two inhabited islands: Villingili Island, located on the north corner and serves as the capital of Northern Huvadhu Atoll (Gaafu Alifu), and Kooddoo Island, located on the south corner and houses a fish processing plant, a local airport, and a resort. Conversely, Miyaru Kandu is located in Vaavu Atoll, in the central area of the Maldives archipelago. On the southern reef stands the Alimatha resort, whereas the northern side is defined by a shallow reef area that begins at a depth of 3 m.

Villingili Kandu is characterized by an oceanic drop‐off that descends to a depth of 30–35 m, and the channel's orientation is towards the east, with an approximate span of 450 m between the northern and southern corners. Miyaru Kandu faces eastward as well; its oceanic drop‐off stands at a depth of 30–32 m with a distance between the two corners of approximately 230 m, and it is characterized by a bathymetric feature within the channel resembling an internal drop‐off, where sharks tend to aggregate with specific current conditions. Channels experience two types of currents depending on the season and tidal cycle: incoming currents occur when water flows from the ocean into the inner atoll; outgoing currents occur when water moves from the inner atoll to the open ocean.

### 2.2 Sampling method

Fieldwork was conducted during the seasonal operational routes of White Wave Maldives onboard of M.Y. *Island Safari 1* and M.Y. *Island Safari 2*, which provided logistical access to Villingili Kandu from January to March and Miyaru Kandu from October to December, as well as from March to the end of April. Between 2022 and 2023, we conducted over 100 dives, totalling approximately 80 h of underwater observations at Villingili Kandu and 20 h at Miyaru Kandu. Dive duration in channel environments is highly variable, ranging from about 20 min to over 1 h depending on current intensity; therefore, we reported total cumulative observation time rather than average dive length.

Laser photogrammetry was performed on five occasions at Villingili Kandu and four at Miyaru Kandu. We selected these dives based on optimal conditions, including high water clarity and low current intensity, whereas on other occasions photogrammetry was not feasible due to the heavy presence of divers from multiple facilities, which altered shark behaviour and limited measurement opportunities to avoid interfering with other divers’ activities. The diving group responsible for collecting biometric measurements, equipped with one device, followed a linear transect from one corner to the opposite corner of the channel, maintaining a consistent direction of navigation along either the oceanic drop‐off or internal drop‐off in Miyaru Kandu. Moreover, whenever diver activity altered shark movement pattern, data collection was suspended to avoid potential bias. This approach ensured a standardised protocol for each survey conducted and minimised the risk of recounting individuals observed in the aggregation during the data collection (Video S1). Eight of the nine instances of biometric data collection were conducted along the oceanic drop‐off of both Villingili and Miyaru Kandu with incoming current, whereas in one instance, measurements were collected following the inner drop‐off in Miyaru Kandu with outgoing current conditions.

During the photogrammetry sessions, we obtained biometric measurements of total length (TL) and pre‐caudal length (PCL) from individuals within aggregations, along with associated metadata (date, time, and dive site). Additionally, we recorded shark behaviours by visual census and extrapolation from videos taken by citizen science, excluding the regular schooling behaviour of grey reef sharks. These behaviours included: mating attempts, which encompass active chasing behaviours, physical interactions and sometimes visible scarring on females (Carrier et al., 1994; Salinas‐de‐León et al., 2017); predation events; chafing behaviours, involving rubbing the body against surfaces or other species to remove parasites, which may also play a role in social or ecological interactions (Gobbato et al., 2024; Pancaldi et al., 2022; Ritter, 2011; Williams et al., 2021); and visits to cleaning stations, specific reef sections or seamounts where sharks and large marine organisms go for parasite and dead tissue removal by cleaner fish, involving altered swimming behaviour or posture to facilitate cleaning (Oliver et al., 2011; Wheeler et al., 2013).

Finally, we compiled opportunistic photographic and video materials taken from divers during recreational dives spanning from 2013 to 2024 at Villingili Kandu. These records, being from citizen‐science operators, were carefully screened and validated by the authors to ensure data quality and consistency. In this context, video characteristics such as duration and field of view varied considerably; therefore, to minimise this bias, all videos were reviewed frame by frame, and the frame with the maximum number of *C. amblyrhynchos* observed (MaxN) was extracted. Indeed, this is a conservative and widely applied metric (Ellis & De Martini, 1995; Priede et al., 1994; Schobernd et al., 2013) for estimating sightings abundance in non‐standardised footage, enabling comparisons while avoiding double‐counting. The selected frame was uploaded to ImageJ and analysed using the ‘Multi‐point’ function to count individual sharks. Each record was collected with associated metadata (date – month, year, dive site, and MaxN value). This approach provides a conservative yet reliable estimate of aggregation size and supports the evidence that Villingili Kandu hosts one of the largest aggregations of *C. amblyrhynchos* recorded in the Maldives.

### 2.3 Laser construction, calibration, and operation

During the two‐year survey period, two custom‐made measurement devices were constructed from an inox stainless steel tube, measuring a total lenghts of 35 cm and 30 cm, respectively. The 35 cm device was used in 2022, while the 30 cm was employed in 2023 and 2024 (Supporting Information S1, Figure S1). Each device was equipped with two green underwater laser pointers (Archon J1 – Archonlight Xiware Technologies Ltd.) mounted laterally on the tube and firmly secured in place using adjustable clamps. A GoPro Hero 9 camera was mounted in the centre of the tube using a mounting bracket attached to the top of the stainless‐steel custom mounting plate welded to the tube. The action camera was used to capture videos at a resolution of 2.7 K at 30 frames per second (fps) in linear mode.

As described by Deakos (2010, 2011), several factors can introduce inaccuracies in measurements, including image distortion due to light refraction, the use of a wide‐angle lens, non‐parallel alignment of the lasers, and parallax error. In the current study, we used a linear field of view ranging from 24 to 49 mm. Moreover, we executed a two‐step calibration process to mitigate the non‐parallel alignment issue of laser pointers, as described accurately in the Supporting Information S1 and S2 (Figure S2).

### 2.4 Laser biometric analysis

To minimise potential measurement inaccuracies associated with non‐perpendicular angles, only photographs in which the body of the shark was fully extended and the laser dots pointed at a 90° angle to the flank were used. Images taken at non‐parallel angles, as per Rohner et al. (2011), were systematically excluded from our analyses. To achieve that, each video was examined using QuickTime Player, and the most suitable frame for each measurement was selected and extracted.

Measurements were determined by importing the images into ImageJ software (version 1.54 g). The number of pixels between the two laser points were counted and then converted using the scaling factor derived from the known distance between the two dots. From each shark, we derived PCL measurements from the tip of the snout to the insertion of the caudal fin, as well as TL measurements from the tip of the snout to the tip of the upper caudal fin. Given that the caudal fin curvature caused by swimming movements can affect TL estimates, we meticulously recorded PCL values for all individuals, following the recommendations of Rohner et al. (2011).

To analyse the relationship between PCL and TL, we used a linear regression model to initially test and quantify their correlation to be able to derive an equation to predict TL from PCL, allowing us to generate predicted total length (PTL) values for individuals in which only PCL could be reliably measured.

**FIGURE 2:**
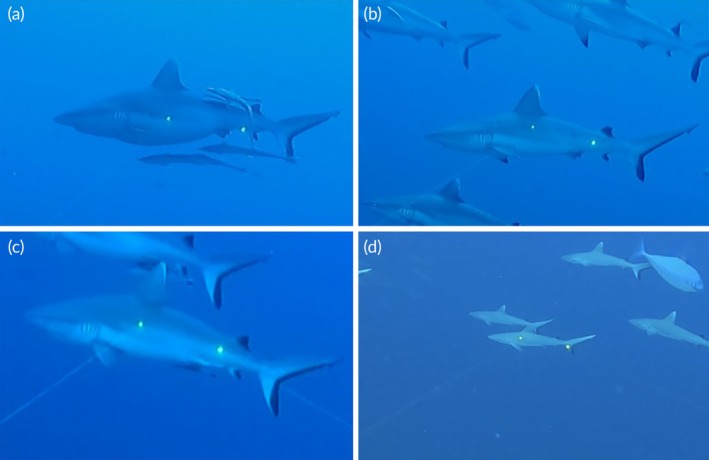
Example of *Carcharhinus amblyrhynchos* individuals measured: (a) mature female with suspected pregnancy; (b) mature female; (c) mature male; (d) young of the year.

Furthermore, for each shark measured, we documented species and sex according to the criteria outlined in Hendon et al. (2013), using the presence of potential mating wounds as an indicator of sexual maturity. Larger female individuals exhibiting a noticeable bulge on the ventral and lateral sides of the body, potentially indicating a pregnancy status (Figure 2a; Acuña‐Marrero et al., 2014; Lea et al., 2015; Robinson et al., 2016; Ramírez‐Macías et al., 2016; Hedrick et al., 2019), were classified simply as mature females, as this condition cannot be confirmed without ultrasound examination or dissection (Sulikowski et al., 2024).

### 2.5 Estimating the size and maturity of *C. amblyrhynchos*

The determination of sexual maturity in male individuals is typically based on the elongation and complete calcification of the claspers, coupled with their ability to fully rotate (Francis & Maolagáin, 2000). However, in our sampling methodology, assessing the calcification state of claspers is challenging and susceptible to potential biases. This challenge is amplified when determining the maturity stage of female individuals, which, in our case, can only be reliably assessed by visual observation of females exhibiting potential signs of pregnancy. Due to variability in the classification of mature and non‐mature individuals reported in studies from different regions around the world (Bradley et al., 2017; Robbins, 2006; Robbins & Renaud, 2015; Smart et al., 2016; Wetherbee et al., 1997), we extensively reviewed the literature, and following the classification provided by Smart et al. (2016), Bradley et al. (2017) and Ebert et al. (2021), we divided measured specimens into three size‐based categories: young of the year (YOY), non‐mature (NM) and mature (M).

The sexual maturity threshold for females was determined based on available literature (Wetherbee et al., 1997; Simpfendorfer et al., 2020; Ebert et al., 2021), with the smallest female showing potential maturity used as reference (Figure 2b). For males, we set the maturity threshold by combining the smallest measured individual exhibiting fully elongated claspers and the criteria outlined by Bradley et al. (2017) (Figure 2c). YOY classification followed Smart et al. (2016) and was further supported by two observations recorded by Anderson and Ahmed (1993), who documented a pregnant female caught in Haa Alif Atoll, Maldives, carrying two embryos measuring 48 and 49 cm TL and a free‐swimming newborn measuring 53 cm TL. Based on this evidence, individuals were classified as YOY if ranging from 45 to 72 cm TL, NM if ranging from 73 to 119 cm TL and M if ≥120 cm TL. Sex was not reported for YOY individuals due to their small size (Figure 2d).

### 2.6 Statistical analyses

All graphs and statistical analyses were produced using RStudio (version 2023.06.2+561). Before analysis, TL data were assessed for normality using the Shapiro–Wilk test. Differences in TL distributions between sites were evaluated using a Wilcoxon rank‐sum test, selected as a non‐parametric alternative when the assumption of normality could not be confirmed. As sampling effort varied among the nine dates, we summarized sex ratio as the average of per‐date female:male (F:M) ratios. Differences in life‐stage composition (YOY, NM, and M individuals) between sites and across sampling dates were assessed using Fisher's Exact Test, a non‐parametric test suitable with small expected frequencies.

## 3 RESULTS

A total of 505 grey reef sharks were measured during dive surveys, specifically 402 in Villingili Kandu and 103 in Miyaru Kandu. Of the total number of grey reef sharks measured, 68.1% (*n* = 274) in Villingili Kandu and 76.7% (*n* = 79) in Miyaru Kandu met the criteria established in this study to minimize parallax error; therefore, 353 individuals were included in the statistical analyses, whereas the remaining were not considered for the statistical analysis or the biometric data collection.

**FIGURE 3:**
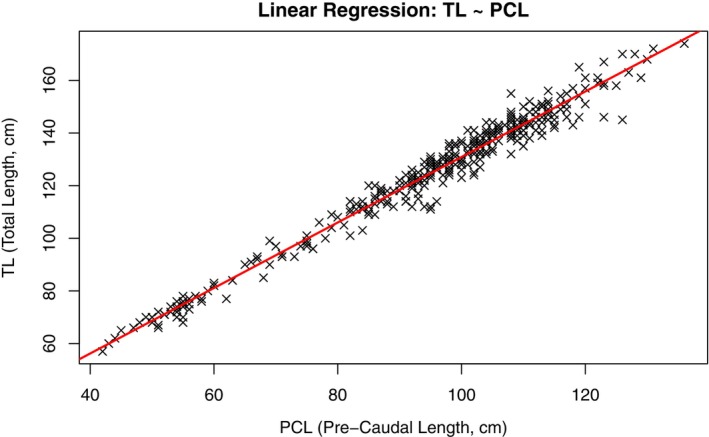
Linear regression of pre‐caudal length (PCL) and total length (TL), in centimetres, of *Carcharhinus amblyrhynchos* (*y* = 6.47 + 1.244 * x). Symbol (X) indicates specimens recorded in both channels.

The linear regression analyses resulted in a strong linear relationship observed between PCL and TL, with a highly significant model (*p* < 0.001, *R* ^2^ = 0.97). These results strongly support using PCL as a robust predictor of TL for *C. amblyrhynchos* (Figure 3).

The aggregation composition revealed differences in both size and sex between the two sites examined. A Wilcoxon rank‐sum test (*W* = 4396, *p* < 0.001) demonstrated a significant difference in TL distributions between the two sites, with Miyaru Kandu exhibiting a wider size range.

**FIGURE 4:**
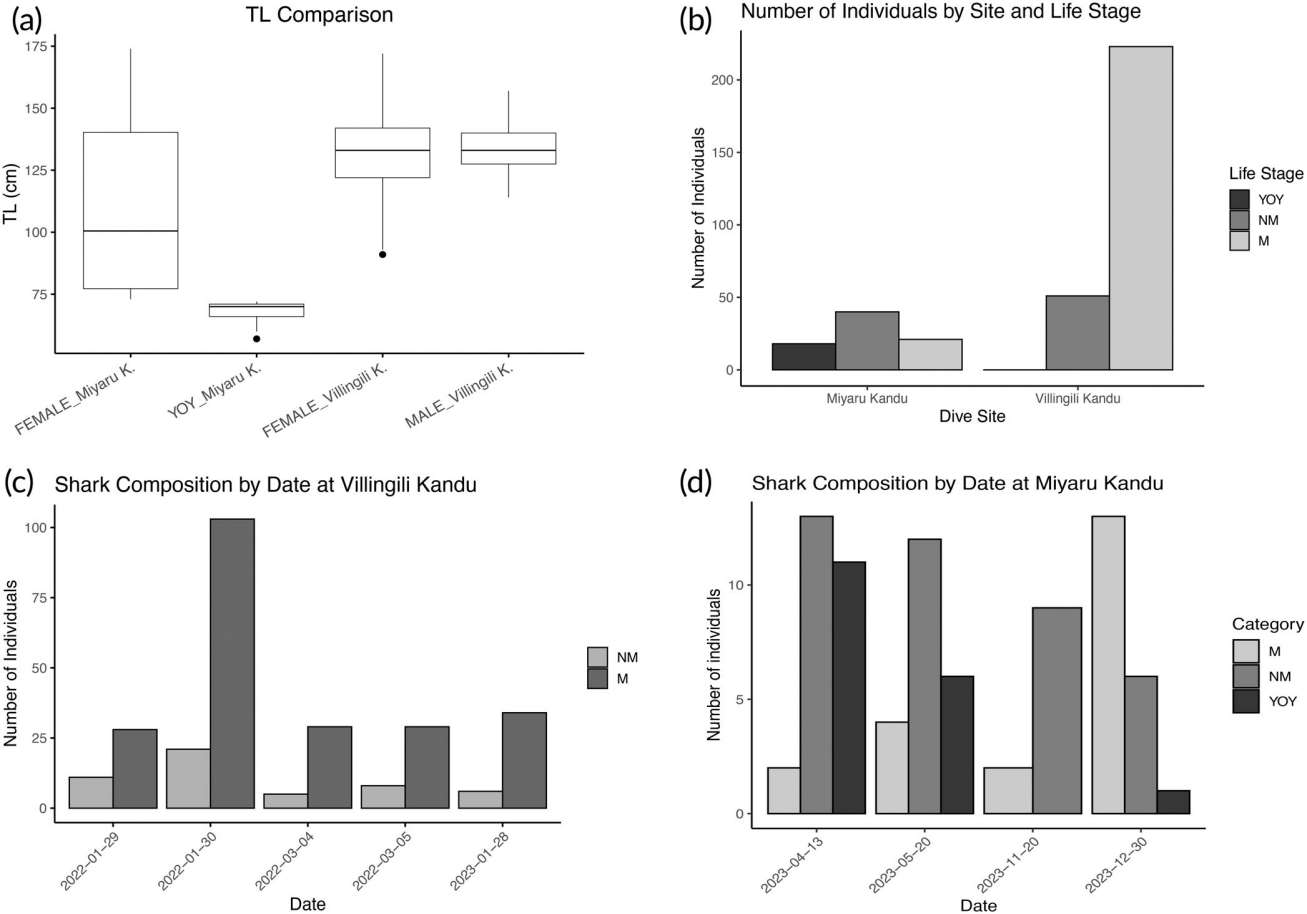
Overview of length and size class distributions of shark in the two sites: (a) Box plot showing total length (TL), in centimetres, of sharks grouped by sex and site; (b) bar plot displaying the number of individuals measured per life stage category (YOY, NM and M) at two dive sites; (c) bar plot showing the measured sharks by life stage category (M and NM) across five sampling dates at Villingili Kandu; and (d) bar plot showing the number of measured sharks by life stage category (M, NM and YOY) across four sampling dates at Miyaru Kandu.

Mature individuals exhibited a mean TL of 137 ± 11.3 cm at Villingili Kandu (*n* = 223) and 144 ± 14.9 cm at Miyaru Kandu (*n* = 21). Non‐mature individuals, instead, had an average TL of 109 ± 9 cm at Villingili Kandu (*n* = 51) and 86.1 ± 15.2 cm at Miyaru Kandu (*n* = 40) (Figure 4a). Notably, YOY individuals (*n* = 18) were observed exclusively at Miyaru Kandu, showing a mean TL of 67.8 ± 4.51 cm (Figure 4a).

Analysis of the sex ratio revealed a significant female bias at both locations. At Villingili Kandu, the average F:M ratio across dates was 12.6 females for every male in the five sampling dates. Conversely, at Miyaru Kandu, the average F:M was not estimable because males were absent on every sampling date.

The temporal assessment of life‐stage composition (Figure 4b) revealed significant variability at Miyaru Kandu across sampling dates (Fisher's exact test, *p* < 0.001), indicating fluctuations in the proportions of YOY, NM and M sharks throughout the sampling periods (Figure 4d). Conversely, the demographic composition at Villingili Kandu remained consistent over time (Fisher's exact test, *p* = 0.499) (Figure 4c).

### 3.1 Behavioural observation

**FIGURE 5:**
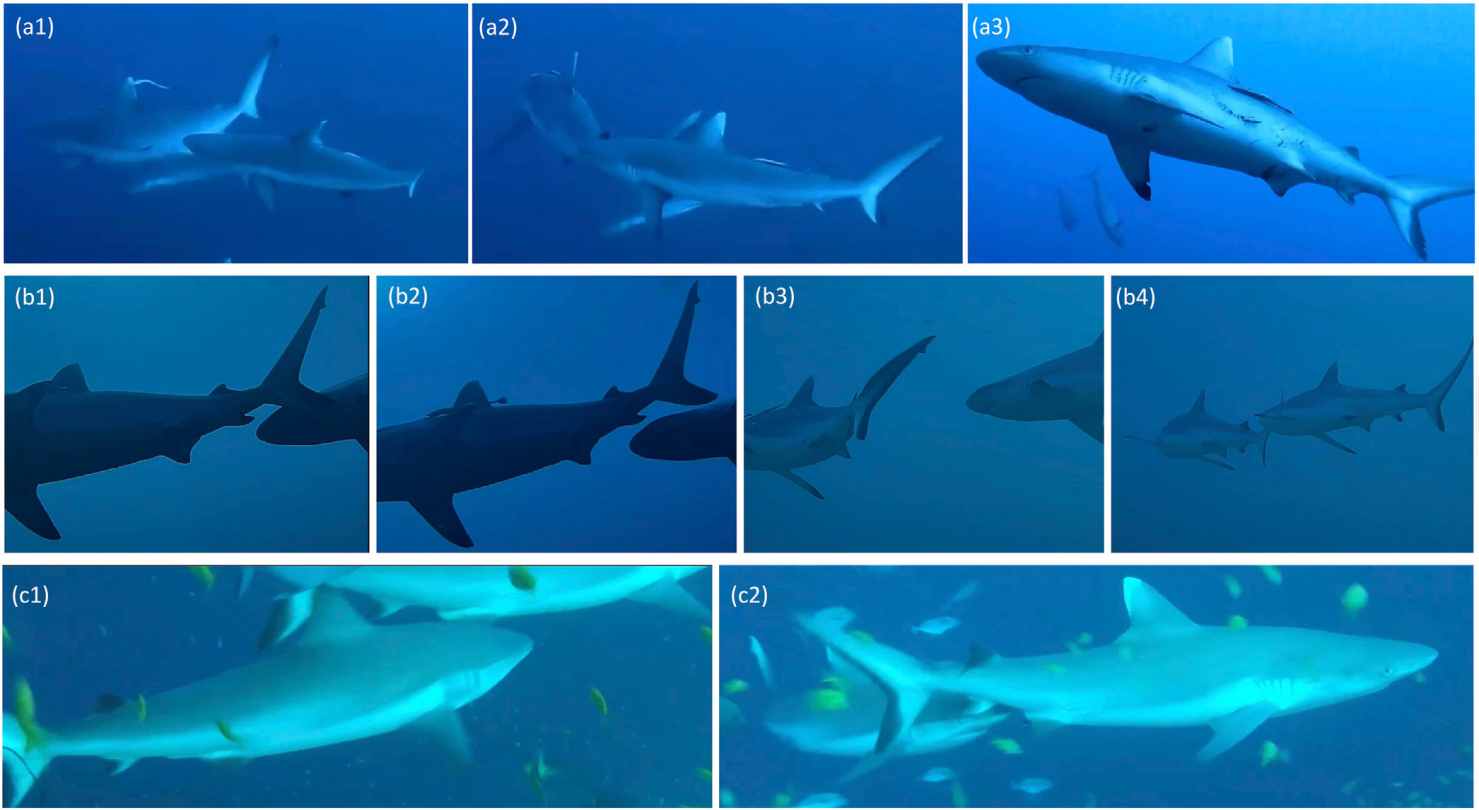
Mating attempts in the two surveyed channels: (a) Sequence from a mating attempt in Miyaru Kandu in January 2022; (a1–a2) Male shark attempting to mate with a female displaying bites from previous attempts/mating; (b1–b4) a male smelling and directly chasing a female with bite marks in the pelvic area in Villingili Kandu, January 2024; (c1–c2) Male smelling and chasing a female in Miyaru Kandu, November 2024.

During our surveys, we documented mating attempts and mating scars on females within the aggregation in January 2022 and December 2024 in Miyaru Kandu, as well as in February 2024 in Villingili Kandu, as illustrated in Figure 5. The presence of mature males in the aggregation during mating periods strongly suggests that the two channels may serve as a breeding ground for the species.

**FIGURE 6:**
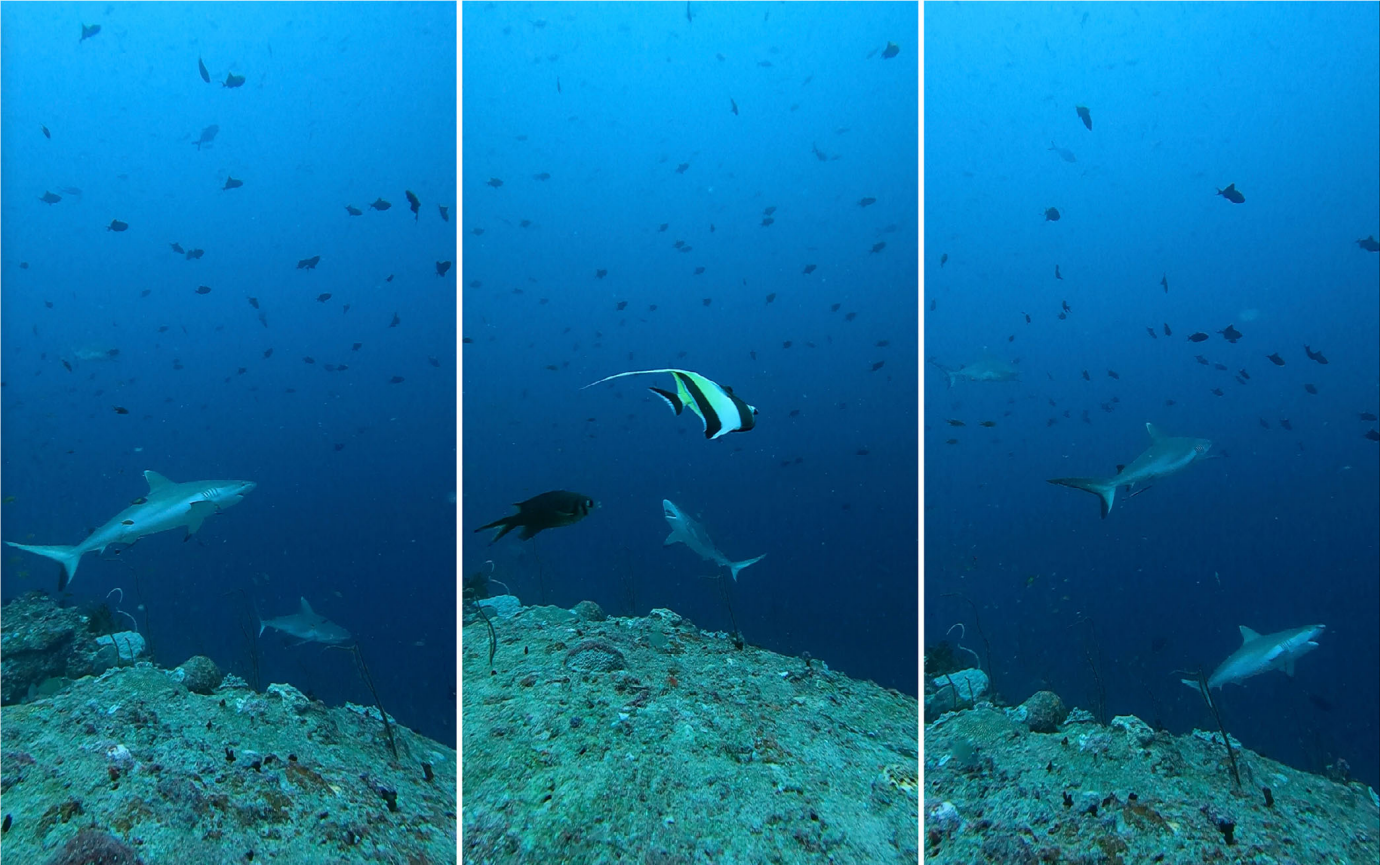
Sequences of grey reef sharks visiting a cleaning station in Miyaru Kandu, Southern Corner, May 2024.

Moreover, in both channels, *C. amblyrhynchos* individuals have been observed visiting and taking advantage of the cleaning station located in the southern corner of the channels (Figure 6). At these spots, they typically approach the cleaning station in a vertical position, facilitating the access for cleaner fish, primarily Labridae, to remove parasites and dead tissue, highlighting the ecological importance of cleaning interactions in maintaining shark health. In addition, in the Villingili Kandu area, instances of chafing behaviour by *C. amblyrhynchos* on *R. typus* have been observed during the year 2022, suggesting that this species may serve as a scraping surface (Gobbato et al., 2024; Williams et al., 2021).

Furthermore, two predation events were documented on separate occasions, specifically on 28 January 2020 and 2 February 2020. In the initial event (Video S2), individuals were observed aiming and focusing on prey located on the channel drop‐off, with the entire sequence lasting 27 s. During the second event lasting 15 s (Video S3), the predatory response of a grey reef shark appeared to be triggered by a predation attempt outside the camera's focus; throughout this event, the aggregation trajectory changed following an attack by a dogtooth tuna (*Gymnosarda unicolor*) on a different target, leading to a rapid change of direction from the group pursuing a different prey.

### 3.2 Assessment of MaxN recorded between 2013 and 2024

MaxN was derived from 11 opportunistic videos recorded by divers between 2013 and 2024 in Villingili Kandu (Table 1). The counts ranged from a minimum of 28 individuals in January 2015 to a maximum of 78 in February 2021. Counts exceeding 60 individuals in a single frame were observed in February 2021, February 2024, and March 2024.

**TABLE 1:** Opportunistic shark aggregation observations at Villingili Kandu (2013–2024) based on guest‐recorded videos.

	Date	Dive site	MaxN per frame
Supp. Info. Figure S3a	February 2013	Villingili Kandu	30
Supp. Info. Figure S3b	January 2014	Villingili Kandu	51
Supp. Info. Figure S3c	January 2015	Villingili Kandu	28
Supp. Info. Figure S3d	March 2019	Villingili Kandu	32
Supp. Info. Figure S4e	January 2020	Villingili Kandu	60
Supp. Info. Figure S4f	March 2020	Villingili Kandu	43
Supp. Info. Figure S4g	February 2021	Villingili Kandu	78
Supp. Info. Figure S4h	March 2022	Villingili Kandu	31
Supp. Info. Figure S5i	February 2023	Villingili Kandu	50
Supp. Info. Figure S5j	February 2024	Villingili Kandu	69
Supp. Info. Figure S5k	March 2024	Villingili Kandu	71

*Note:* Sampling dates are listed by month and year. The MaxN frame indicates the maximum number of sharks visible in a single image from each video. Representative frames were analysed with the multi‐point function in ImageJ (Figures S3–S5).

These values represent conservative abundance estimates, as they are based on the maximum shark number observed simultaneously in a video frame, avoiding repeated counts of the same individuals within a recording.

## 4 DISCUSSION

Understanding the ecological drivers behind shark aggregations is essential to develop evidence‐based conservation strategies and management of key habitats for these animals. In this context, the preliminary findings presented in this study provide a baseline for further research on grey reef sharks’ aggregation dynamics in the Maldives.

Several factors must be taken into consideration to explain the aggregations of *C. amblyrhynchos* as this species is known for its strong residency and site fidelity (Espinoza et al., 2015; Micarelli et al., 2024; Vianna et al., 2013). Generally, adult males exhibit greater mobility than females, likely due to reproductive migration to potential mating grounds in different areas that could promote genetic dispersion, as previously documented in populations of the Great Barrier Reef (Bonnin et al., 2019; Lesturgie et al., 2023; Momigliano et al., 2017). The average sex‐ratio recorded in both channels highlighted a strong female bias with 12.6 females recorded per male in Villingili Kandu and no males recorded in Miyaru Kandu, suggesting that these two aggregations are mainly formed by female individuals; however, additional data is required to investigate what are the environmental or hormonal factors that determine the beginning of the mating season inside the aggregation. Nevertheless, we recorded evidence of reproductive activities, including records of mating attempts and mating scars on females in both aggregations, particularly in January 2022 and December 2024 in Miyaru Kandu, and February 2024 in Villingili Kandu (Figure 5), suggesting that these two channels may act as reproductive habitats, potentially serving as mating grounds for mature individuals.

**FIGURE 7:**
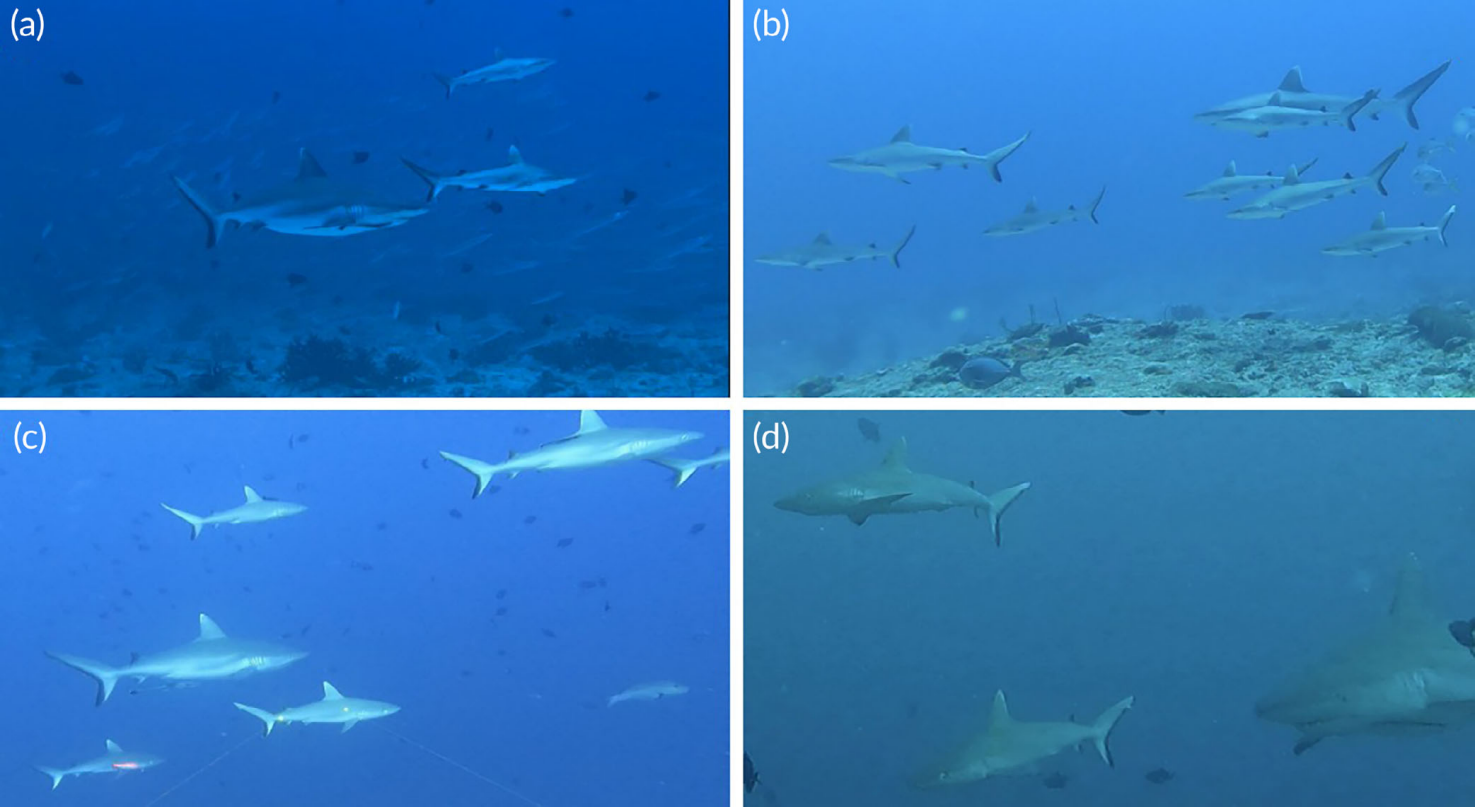
Grey reef sharks – juveniles and YOY individuals schooling with adults in Miyaru Kandu in: (a) April 2023, (b) May 2023, (c) May 2024, and (d) December 2023.

We recorded the presence of YOY in Miyaru Kandu and the absence of YOY individuals in Villingili Kandu, which does not necessarily exclude the possible presence of a nursery area in proximity to the aggregation. Current literature indicates that, in addition to sex segregation, there also exists a segregation based on size, where juvenile individuals tend to aggregate in different size groups compared to adults, as observed in species such as dusky sharks (*Carcharhinus obscurus*) and sandbar sharks (*C. plumbeus*) in the Mediterranean Sea (Bigal et al., 2024) and Western Australia (Pember et al., 2023), as well as whale sharks (*Rhincodon typus*) in the Gulf of California and the Maldives (Ketchum et al., 2012; Riley et al., 2010), blacktip reef sharks (*Carcharhinus melanopterus*) in French Polynesia (Mourier et al., 2013) and silky sharks (*Carcharhinus falciformis*) in the Pacific Ocean (Kindong et al., 2022). Despite this evidence, in Miyaru Kandu, we recorded YOY and NM individuals swimming alongside adult individuals on several occasions, an uncommon behaviour for other reef species (Matich et al., 2017) (Figure 7). YOY individuals were consistently observed throughout 2023, and archival records confirm their recurrent presence in prior years, demonstrating sustained use of the channel as a nursery habitat (Supporting information, Video S4). Together, these observations, in accordance with Heupel et al. (2007), lead us to hypothesise that this specific site may function as a nursery area for *C. amblyrhynchos* in this region.

Beyond reproductive behaviours, another significant factor that may influence the abundance of grey reef sharks is the presence of low trophic order fish biomass aggregations, which have been demonstrated to attract predators, especially during their spawning events (Tickler et al., 2017; Rhodes et al., 2019). In Villingili and Miyaru Kandu, we consistently recorded the presence of numerous potential prey species from the Caesionidae, Carangidae*,* and Priacanthidae families (Figure S6). These species are known to form group spawning events, providing a readily available food source. We recorded predation events on two different occasions (Videos S2 and S3), suggesting, as Mourier et al. (2016) noted, that sharks may benefit from the presence of these schools as a food source that might reduce the need for long foraging trips outside the channel.

Additionally, hydrodynamic conditions further support aggregation behaviour, as the presence of strong currents can reduce the need for continuous swimming, as seen in French Polynesian atoll channels with grey reef sharks aggregating along oceanic drop‐offs or in specific areas within the channel to take advantage of updraft areas created by these bathymetric features in conditions of incoming and outgoing currents to rest and move with minimal energy expenditure (Laurioux et al., 2025; Papastamatiou et al., 2021). Similarly, we recorded consistent patterns at both our study sites, wherein *C. amblyrhynchos* aggregated along channel drop‐offs during incoming currents. Under outgoing current conditions, sharks were seen moving in two different areas between sites: in Miyaru Kandu, they were observed aggregating inside the channel, where the bathymetry of the inner drop‐off creates an alternative updraft area; in contrast, in Villingili Kandu, the aggregation moved below the drop‐off, potentially to avoid unfavourable current conditions and optimise energy expenditure (Video S5). Although considered obligate ram ventilators (Dapp et al., 2015; Tickler et al., 2017), recent observations have documented *C. amblyrhynchos* individuals aggregating at isolated reef ledges in the Republic of Seychelles, where they were observed resting and unresponsive (Bullock et al., 2024). Similar behaviour has been recorded in specific locations in the Maldives (De Maddalena, 2023; Parmegiani, pers. obs., 2025; Figure S7). Although such behaviour was never recorded within channels, these observations suggest that this species may use sheltered reef areas, in addition to updraft regions, to rest and potentially avoid predators.

In both channels, we observed grey reef sharks visiting cleaning stations near the oceanic drop‐off (Figure 6). Although we do not have sufficient data to determine if this behaviour is favoured by determinate tidal or current conditions, our sightings align with patterns observed in other regions (Wheeler et al., 2013) and among other elasmobranch species, which use specific cleaning stations, coral formations or particular areas of the reef (O'Shea et al., 2010; Oliver et al., 2011, 2019; Carpenter et al., 2024). We recognise these specific areas as aggregation hotspots within the channels, where mutualistic interactions between cleaners and hosts may promote site fidelity.

In comparison to previous studies from the Maldives, shark sightings at Villingili Kandu were notably higher. SharkWatch surveys, which provided baseline data on reef shark distribution and abundance across the Maldives, reported that favourable sites for shark sightings typically averaged *n* = 29 individuals (Sattar et al., 2013). Conversely, our surveys documented larger aggregations at Villingili Kandu, with photographic evidence collected from several videos showing MaxN of up to 78 *C. amblyrhynchos* in a single frame (Figure S4f). Considering that this count is a conservative estimate, as it does not include all individuals observed in the video footage (Videos S6 and S7), these findings through different years indicate that Villingili Kandu currently hosts the largest recorded aggregation of *C. amblyrhynchos* in the Maldives.

Moreover, to ensure consistent biometric data retrieval in different field conditions, we derived all PTL values from a linear regression model based on the measured values of PCL and TL. The model demonstrated a strong fit, indicating that PTL can be a reliable predictor of TL, especially in cases where measurements may be influenced by tail flexion or when entire body measurements are impractical. This is particularly significant in environments such as channels, where the strong current creates significant challenges for video capture, especially for less experienced divers. Moreover, it can be very useful for non‐scientist contributors who are playing an expanding role in citizen science efforts related to conservation initiatives (Araujo et al., 2020; Roff et al., 2016; Siena et al., 2025; Whitehead et al., 2025), as this model may serve as a reliable tool for validating citizen science data and enhancing its scientific utility.

In conclusion, although our data provide an initial overview of two shark aggregation sites, further surveys are needed. These locations clearly represent important habitats similar to those super habitats recently demonstrated on grey reef shark aggregations in French Polynesia, where a single channel can serve multiple purposes, including foraging, refuge, mating and parturition for over 500 resident individuals (Papastamatiou et al., 2025). These findings should be considered for further protection and included within recent ISRA initiatives to identify sensible areas for elasmobranchs in the region. Moreover, recent ID techniques used to identify individuals from different species of requiem sharks could be integrated to better evaluate the site fidelity and movement in the channels (Lionnet et al., 2025; Micarelli et al., 2024). Merging these data with diver observations and local community knowledge facilitates the identification of important shark aggregation habitats, enhancing targeted conservation strategies, supporting the development of locally adapted management measures and contributing to regional efforts ensuring the long‐term viability of reef shark populations.

## AUTHOR CONTRIBUTIONS

Conceptualization: A. P., J. G., D. A. W.; validation: D. S., S. M., Y. R., P. G.; formal analysis: A. P., J. G., D. A. W., I. Y.; investigation: A. P., J. G., I. Y., D. A. W.; writing – original draft preparation: A. P., J. G., D. A. W., Y. R.; writing – review and editing: A. P., J. G., D. S., S. M., P. G.; supervision: D. S., S. M., Y. R., P. G. All authors have read and agreed to the published version of the manuscript.

## FUNDING INFORMATION

This research received no specific grant from any funding agency in the public, commercial or not‐for‐profit sectors.

## Supporting information


**Data S1.** Supporting Information.

**Video S1.** Example of a laser photogrammetry session on Villingili Kandu grey reef shark aggregation.

**Video S2.** Predation event observed involving a prey located on the reef at the channel drop‐off.

**Video S3.** Change in behavior triggered by a predation attempt outside the camera’s field of view.

**Video S4.** Supporting evidence of observations on the presence of YOY grey reef sharks in the study area.

**Video S5.** Shark aggregation moving below the drop‐off in Villingili Kandu in conditions of outgoing current, potentially to avoid unfavorable current and optimize energy expenditure.

**Video S6.** First supporting evidence for Villingili Kandu as the putative largest recorded aggregation of *C. amblyrhynchos* in the Maldives.

**Video S7.** Second supporting evidence for Villingili Kandu as the putative largest recorded aggregation of *C. amblyrhynchos* in the Maldives.
