# Supplementary material for: Insights into the structure of grey reef shark aggregation, Carcharhinus amblyrhynchos (Bleeker, 1856), in two distinct channels of the Maldivian archipelago, Indian Ocean
Source: J Fish Biol. 2026 Mar 3;108(4):1347–60. doi: 10.1111/jfb.70337 (PMC13193483; doi:10.1111/jfb.70337)
Supplement: Supplementary file 1 — Data S1. Supporting Information. [file JFB-108-1347-s003.docx]

**Supporting information**

**S1. Calibration of the laser photogrammetry system device**

First, the calibration was performed outside the water by measuring a fixed reference object at distances of 5, 10, and 25 meters and adjusting the laser positioning until accurate measurements were achieved. Subsequently, we conducted three underwater calibrations in a lagoon with a sandy bottom. To establish reference distances, we used a rope marked at 3, 6, 9, and 12 meters, moving down to a depth of 10 meters. A calibration object, comprised of a plastic structure with a total length of 75 cm, featured two fixed reference points at a distance of 30 cm. Two operators worked underwater, one ensuring the object's stability during measurements while the other moved to the designated distances for the calibration process. The measurements were repeated 3 times at each distance to assess the precision and accuracy of the measurements. The image clarity was satisfactory at distances of 3 to 6 meters, but at 9 meters, the laser beams began to lose definition due to the reduced power of the device. In contrast, at 12 meters, the laser dots became almost invisible on the surface of the reference object.

Finally, to minimize parallax error, we followed the procedure outlined in Rohner et al. (2011), conducting measurements of the 75 cm reference object at varying angles of 0°, 20°, and 45° using a diving compass. Subsequently, we processed the captured images with ImageJ, from which we derived an estimated error for each angle.

Underwater data collection was conducted by a diver who used the laser device to point the two lasers directly at the region situated between the gills and the caudal peduncle of the sharks. This designated area serves to prevent potential errors associated with tail flexion and to avoid projecting the lasers directly into their eyes, which can deter and disturb their natural behaviors.


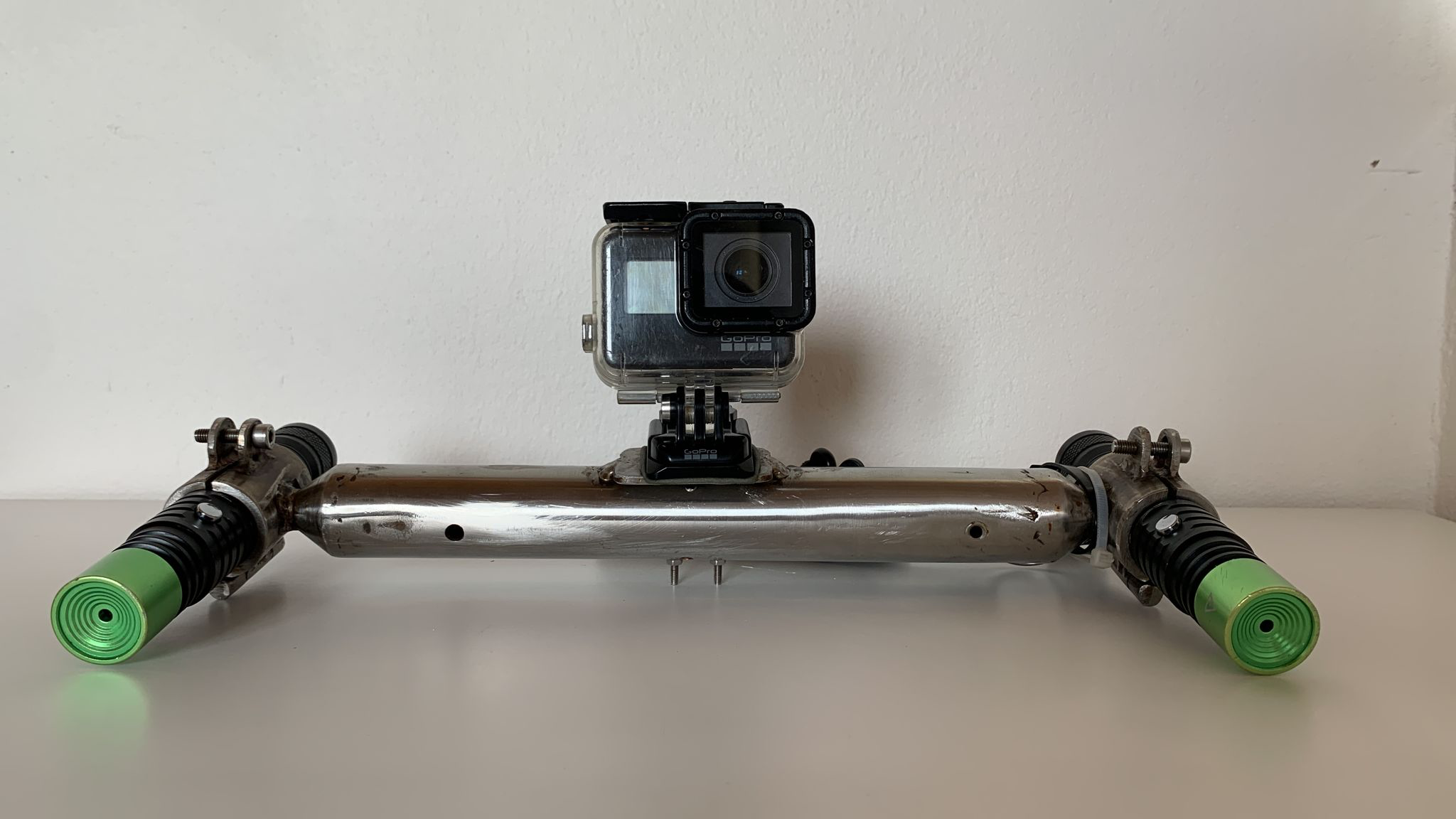


**Figure S1.** Laser photogrammetry device used by divers collecting biometric data


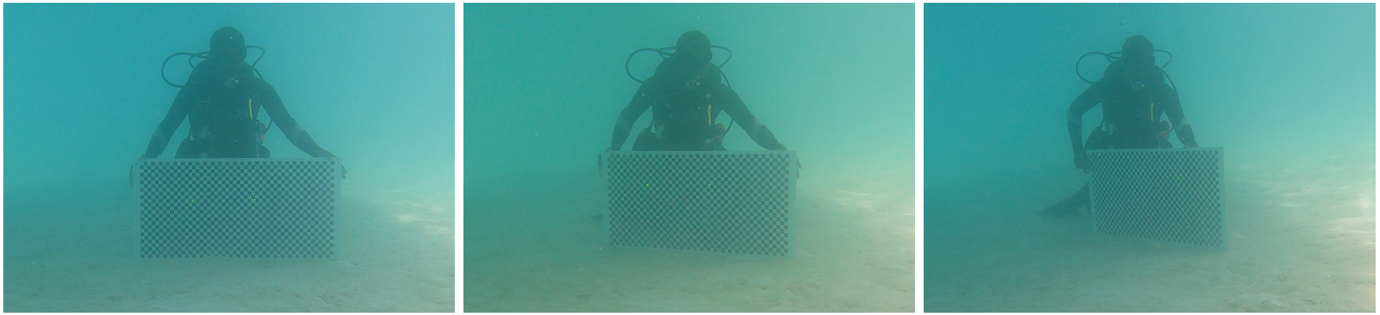


**Figure S2.** Calibration at 0°, 10°, 45°

**S2. Calibration results**
The device calibration indicated a distance between the two laser rays of 35.4 ± 0.3 cm for the first device and 30.4 ± 0.5 cm for the second device (Table 1).

**Table S1.** Laser photogrammetry calibration test result

| *Laser calibration at 35 cm* | | | | |
| --- | --- | --- | --- | --- |
| **Distance to the object** | **Laser Distance 1** | **Laser Distance 2** | **Laser Distance 3** | **Mean ± SD** |
| 3 mts | 35.0 | 35.0 | 35.0 | 35.0 ± 0.0 |
| 6 mts | 35.3 | 35.2 | 35.3 | 35.2 ± 0.1 |
| 9 mts | 36.0 | 35.7 | 35.9 | 35.9 ± 0.2 |
| Combined mean ± SD | | | | 35.4 ± 0.3 |
|  | | | |  |
| *Laser calibration at 30 cm* | | | | |
| **Distance to the object** | **Laser Distance 1** | **Laser Distance 2** | **Laser Distance 3** | **Mean ± SD** |
| 3 mts | 30.0 | 30.0 | 30.0 | 30.0 ± 0.0 |
| 6 mts | 30.2 | 30.4 | 30.5 | 30.4 ± 0.2 |
| 9 mts | 30.7 | 31.0 | 30.9 | 30.9 ± 0.2 |
| Combined mean ± SD | | | | 30.4 ± 0.3 |

**Table S2.** Parallax error assessment

| *Parallax Error at 35 cm* | | | |
| --- | --- | --- | --- |
| **Distance to the object** | **Rotation angle** | **Laser distance** | **Error %** |
| 3 mts | 0° | 35.0 cm | 0% |
| 3 mts | 10° | 35.7 cm | +2% |
| 3 mts | 45° | 46.0 cm | +31.4% |
| *Parallax Error at 30 cm* | | | |
| **Distance to the object** | **Rotation angle** | **Laser distance** | **Error %** |
| 3 mts | 0° | 30.0 cm | 0% |
| 3 mts | 10° | 32.7 cm | +9.1% |
| 3 mts | 45° | 41.6 cm | +38.7% |

Images captured from different angles showed a 2% increase in the known distance between laser dots at a 10° angle. Additionally, there was a 31.4 % increase relative to the known distance in photos taken at 45° (Table 2). The shorter laser device produced larger errors, with 9.1% at 10° and 38.4% at 45°.

In our study, we employed two distinct laser photogrammetry setups (Figure 1S): one at a distance of 35 cm and the other at 30 cm. Error increased with angular displacement (Table 2), especially in the 30 cm setup, reaching 38.7% error at a 45° rotation angle, compared to 31.4% at the same angle for the 35 cm setup. This difference can be attributed to the narrower triangulation baseline, which amplifies angular distortion and measurement inaccuracy. However, considering the optimal field of view of grey reef sharks (Lisney et al., 2007), making them particularly sensitive to disturbances caused by laser photogrammetry as seen in the video collected during the surveys (see Supporting Information 2), we opted for the smaller photogrammetry device. This design choice helped in minimize visual disturbance and consequent disruptions of the aggregation and to avoid accidental pointing of the laser in the sharks’ line of sight during data collection. Furthermore, the compact setup also proved more effective for close-range measurements, particularly of young-of-the-year (YOY) individuals, and better suited for strong currents encountered in Maldivian channel environments.

**S3. Video analysis of Villingili Kandu aggregation**

**
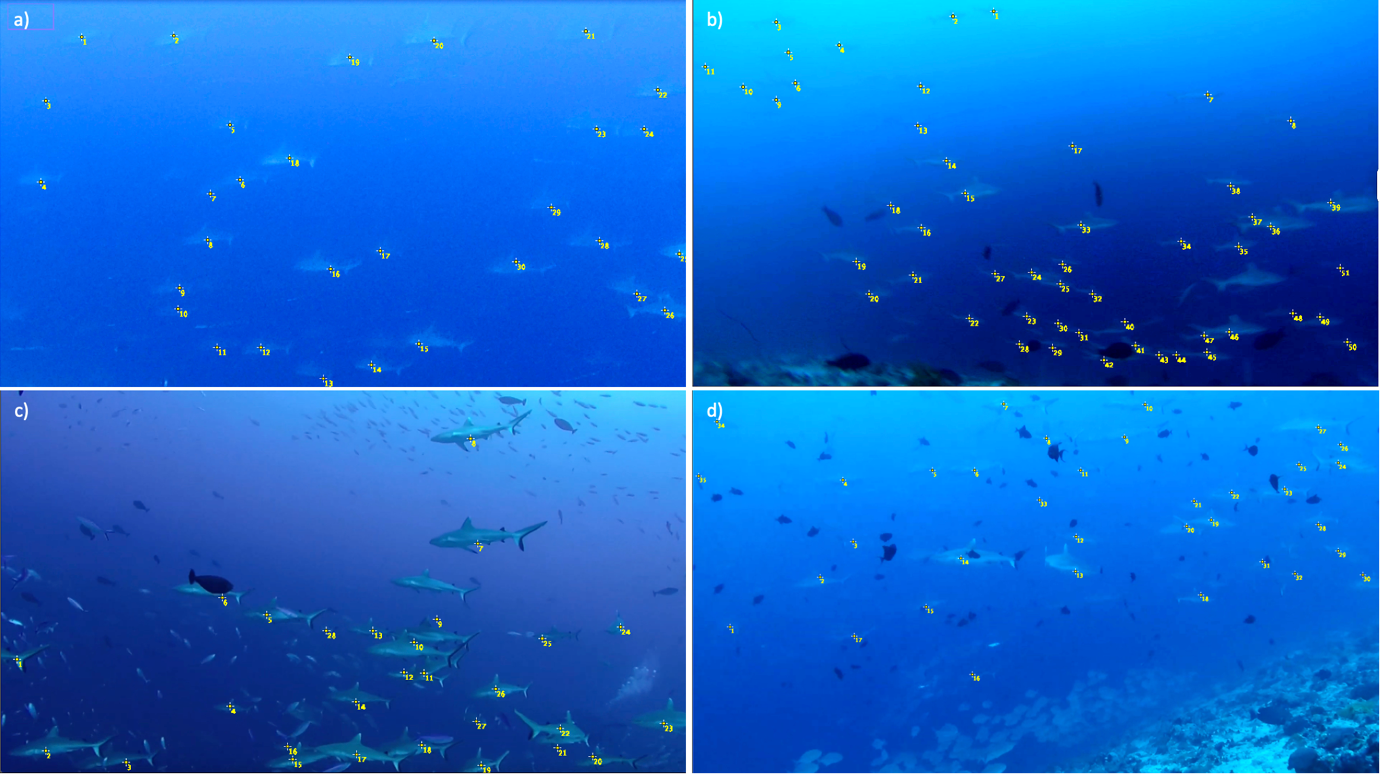
**

**Figure S3. Visualization of MaxN 2013-2019:** (a) MaxN = 30, Villingili Kandu, February 2013; (b) MaxN = 51, Villingili Kandu, January 2014; (c ) MaxN = 28, Villingili Kandu, January 2015; and (d) MaxN = 32, Villingili Kandu, March 2019.

**
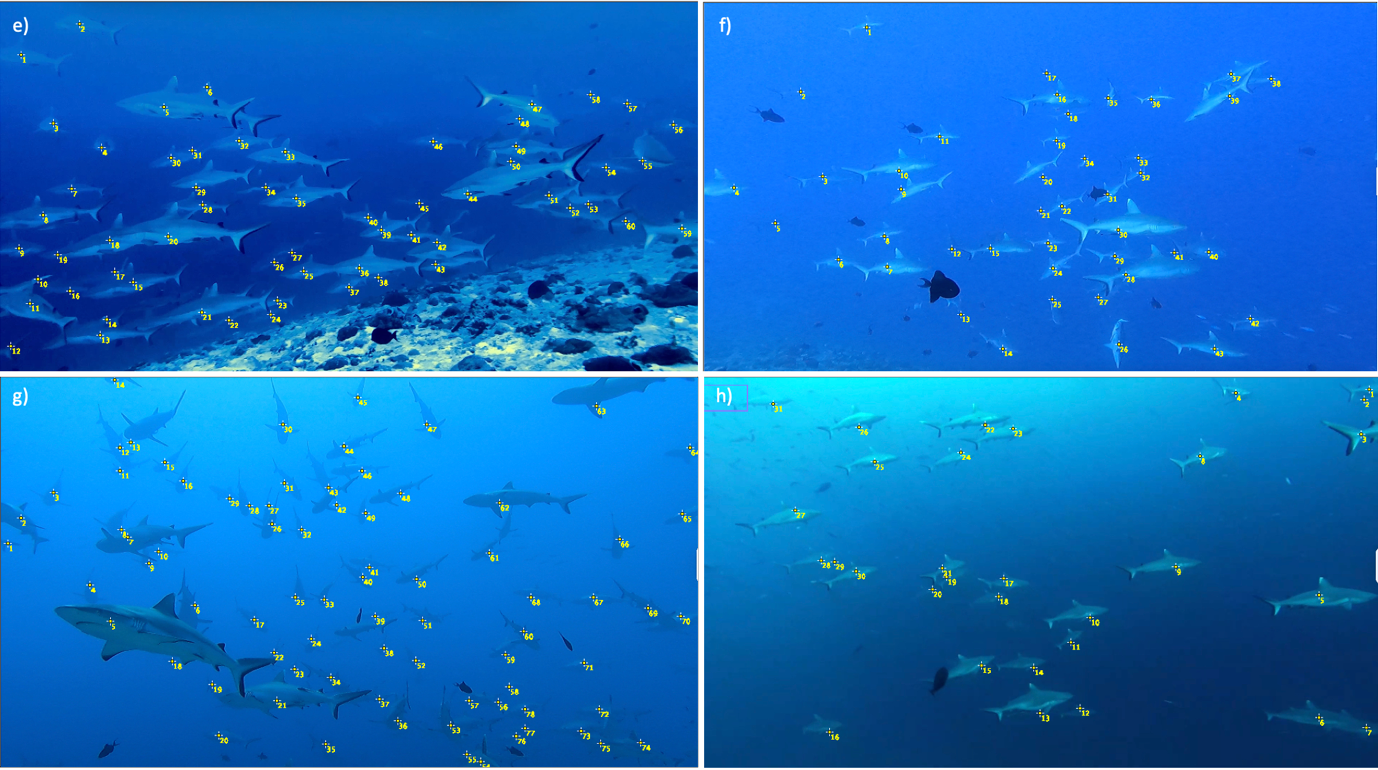
**

**Figure S4. Visualization of MaxN 2020-2022:** (e) MaxN = 60, Villingili Kandu, January 2020; (f) MaxN = 43, Villingili Kandu, March 2020; (g) MaxN = 78, Villingili Kandu, February 2021; and (h) MaxN = 31, Villingili Kandu, March 2022.

**
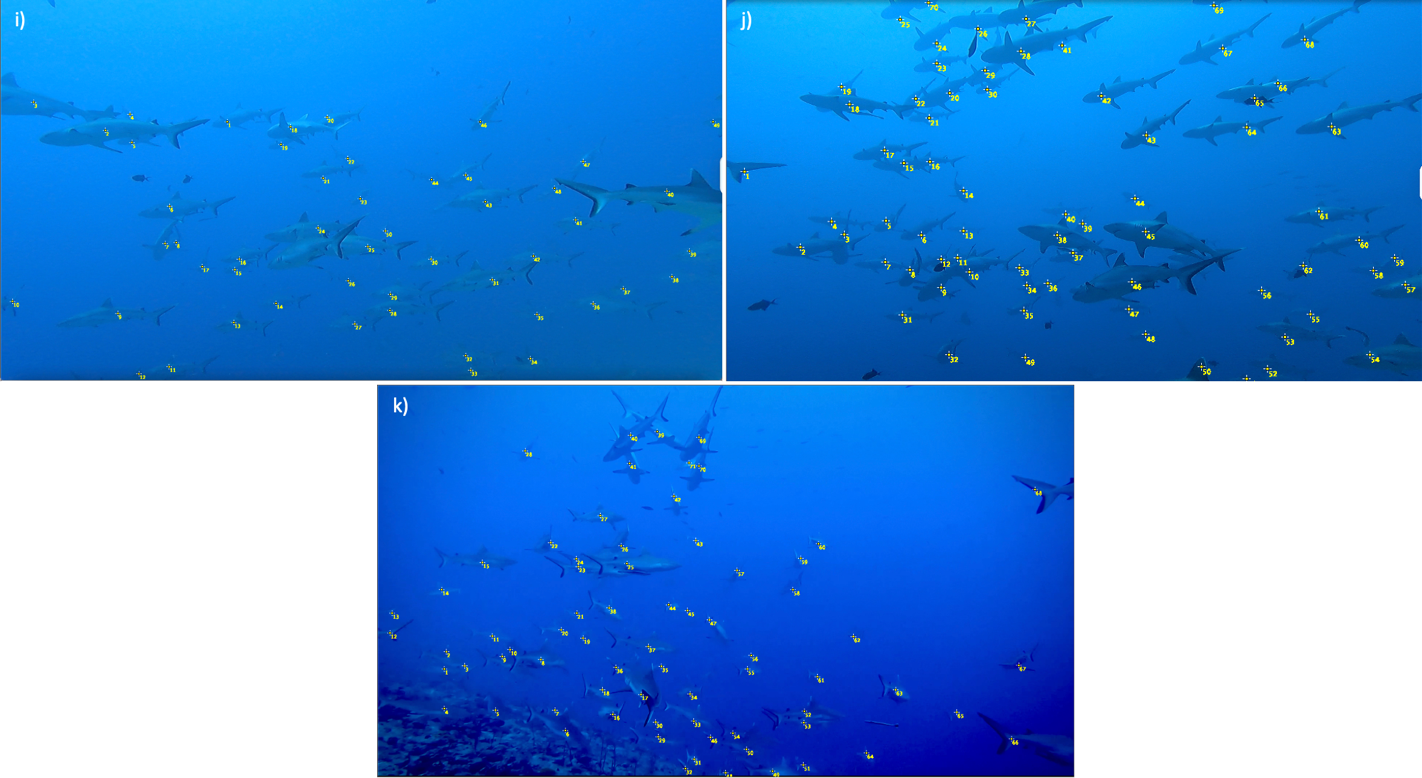
**

**Figure S5. Visualization of MaxN 2023-2024:** (i) MaxN = 50, Villingili Kandu, February 2023; (j) MaxN = 69, Villingili Kandu, February 2024; and (k) MaxN = 71, Villingili Kandu, March 2024.

**
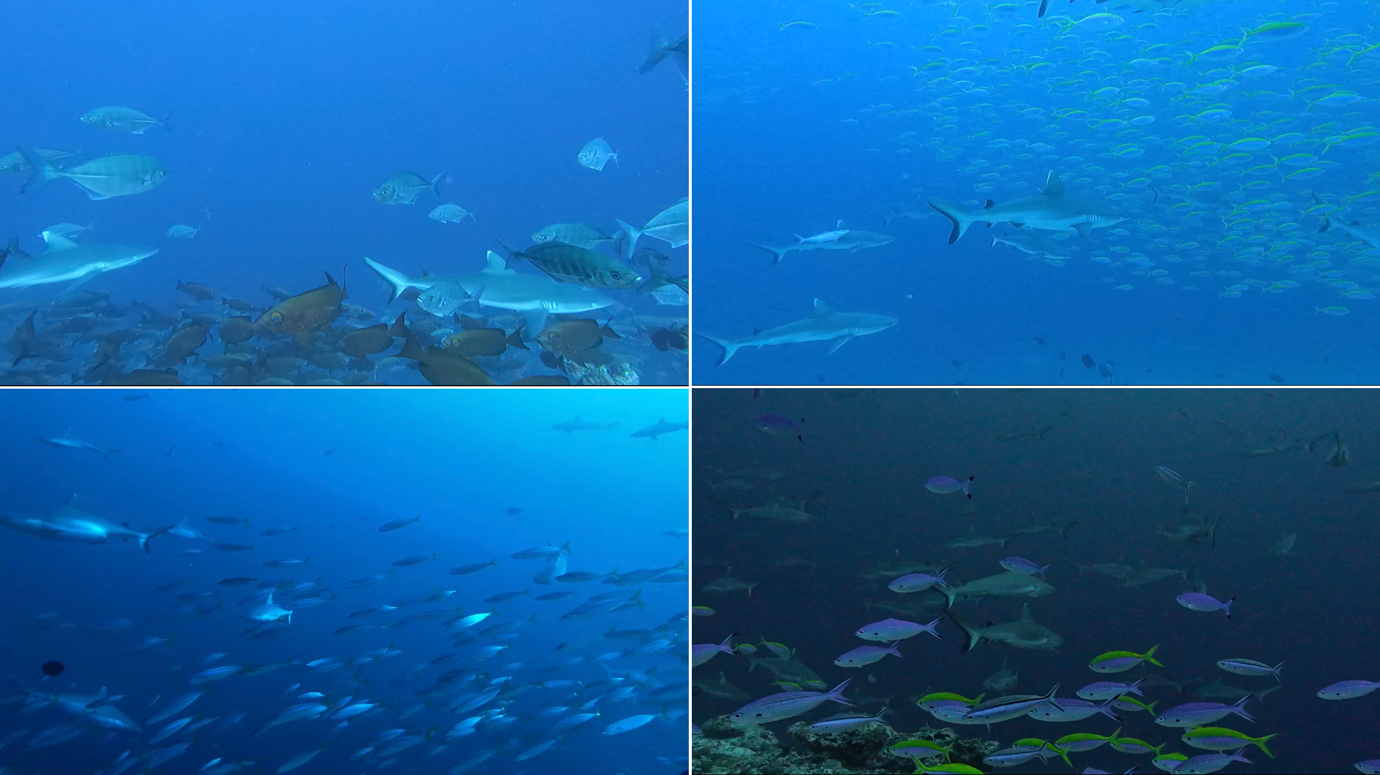
**

**Figure S6.** Grey reef sharks in Miyaru Kandu and Villingili Kandu, swiming alongside groups of *Caesionidae, Carangidae, and Priacanthidae*. This species is known to prey on the latter and serves as a potential food source for the aggregation.

**
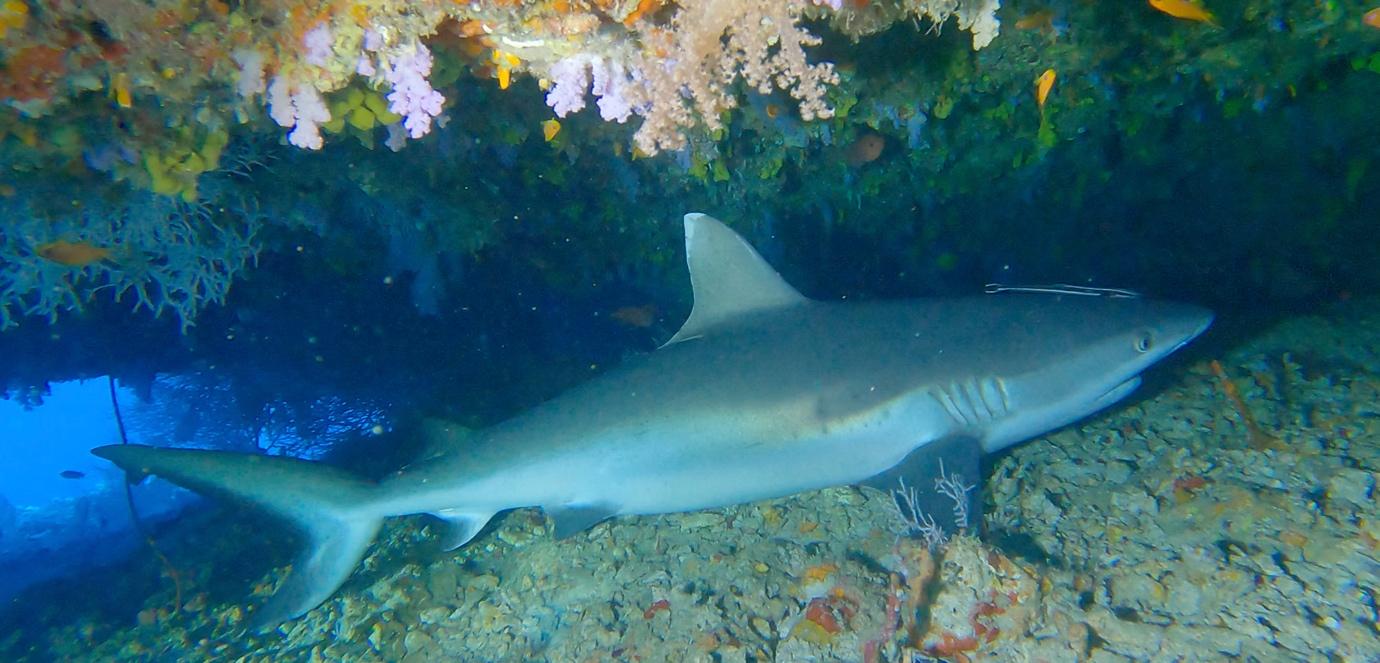
**

**Figure S7.** Grey reef shark resting under a reef edge in Raa Atoll, Republic of Maldives.
